# Phylogeographic history of *Parthenocissus* (Vitaceae) in North America based on chloroplast and nuclear DNA sequences

**DOI:** 10.3389/fpls.2025.1521784

**Published:** 2025-06-25

**Authors:** Di Wu, Ying Meng, Jun Wen, Ze-Long Nie

**Affiliations:** ^1^ Key Laboratory of Plant Resources Conservation and Utilization, College of Biological Resources and Environmental Sciences, Jishou University, Jishou, Hunan, China; ^2^ Department of Botany, National Museum of Natural History, Smithsonian Institution, Washington, DC, United States

**Keywords:** North America, genetic diversity, *Parthenocissus*, phylogeography, population genetic structure

## Abstract

Knowledge of historical distribution and postglacial phylogeographic evolution of plants is important for better understanding their current distribution, population structure and potential fate in the future. Surprisingly, little is known about the post-glacial recolonization history of lianas that are widely distributed in the deciduous or mixed deciduous-evergreen forests in North America. Here, we conducted a phylogeographic study on 47 populations with 398 individuals from the North American *Parthenocissus* using both chloroplast and nuclear DNA sequences data. A high level of genetic diversity is observed among *Parthenocissus* populations in North America, with 66.45% of cpDNA and 92.78% of nrDNA genetic variation present within populations. The North American *Parthenocissus* is roughly grouped into three main lineages with a south to north trend of decline in genetic diversity, which may have been isolated and diverged due to climatic and geographic environmental influences since the late Miocene. Our results indicate that a wide range of gene flow and frequent hybridization are occurring among the *Parthenocissus* populations and the Edwards Plateau, the southern Appalachian Mountains and the Atlantic coastal plains are their possible glacial refugia in eastern and southern North America. The results for *Parthenocissus* represent the first phylogeographic analysis of a major lineage of temperate woodland climbers in North America and support the importance of long-distance dispersal events leading to extensive hybridization and gene flow during the post-glacial migration of this plant lineage.

## 1 Introduction

The climate and environment on Earth have fluctuated dramatically since the Cenozoic era (Hewitt, 2000). Climate oscillations, topographic and hydrological barriers have influenced the location of suitable habitats and the migration of plant populations (Critchfield, 1984). Therefore, knowledge of the historical distribution and post-glacial evolution of plants may provide insights into the possible impact of climate change on the future ranges.

Phylogeography aims to relate evolutionary processes to spatial, temporal and environmental factors in an effort to understand past and present biodiversity (Avise, 2000). Over the last few decades numerous phylogeographic studies have shown that most of the unglaciated geomorphological environment in eastern North America (unlike Europe, with its east-west mountain ranges) is defined by the Appalachian Mountains extending from north to south, and the gradual transition between ecosystem types (Taberlet et al., 1998; Petit et al., 2005; Eckert et al., 2008). Many studies have supported the presence of multiple glacial refugia in eastern North America, such as the Appalachians, and the Atlantic and Gulf coasts (Godbout et al., 2005; Li et al., 2013; Barnard-Kubow et al., 2015). By analyzing 396 published studies, Soltis et al. (2006) evaluated at least six models to explain the major phylogeographic patterns of the unglaciated eastern North America and contemporary range discontinuities. Most of these geographic barriers were formed in the Appalachian Mountains, and/or on both sides of the Apalachicola, Tombigbee, or Mississippi rivers, as well as in coastal areas along the Atlantic Ocean and the Gulf of Mexico (Jaramillo-Correa et al., 2004; McLachlan et al., 2005; Li et al., 2013). Most phylogeographic studies show that there also exist refugia (Culver et al., 2000; Barrow et al., 2015). Barrow et al. (2015) discovered through analyzing genetic structure in a chorus frog species complex that central Texas represented a refugium from which populations expanded via multiple routes.

Despite numerous phylogeographic studies on woody plants, well-delineated glacial refugia generally shared by most species have not been conclusively identified (Ruiz-Sanchez and Ornelas, 2014; Zinck and Rajora, 2016). Proposed refugial locations include the Gulf Coast, the Atlantic Coast, the Ozark Plateau, the Lower Mississippi River Valley, the Edwards Plateau, the Appalachians, and interior areas near ice sheets (e.g., the Labrador region in eastern Canada, which is near the edge of the Laurentide Ice Sheet, the Great Lakes region in northern United States, and the northern part of the Rocky Mountains near the Cordilleran Ice Sheet (Barnard-Kubow et al., 2015; Jaramillo-Correa et al., 2009; Barrow et al., 2015; Soltis et al., 2006). During the Pleistocene (2.4×10^6^ yr(myr)-10,000 yr ago), at least six glacial events occurred, affecting the natural and biological environments in the Northern Hemisphere (Cox et al., 1977). In North America, the Wisconsin Glaciation began 120,000 yr ago and ended approximately 8,000 yr ago (Davis, 1983). At the peak of the glacial period, the ice sheet extended southward to 40°N in the eastern North America. Around 18,000 yr ago, as the Wisconsin ice sheet started to recede, the species that had survived in the ice-free refuges began to migrate northward to the habitats that had previously been covered by glaciers (Griffin and Barrett, 2004). Recently, molecular markers have been used to investigate liana species whose ranges span both formerly glaciated and unglaciated portions of eastern North America. Pollefeys and Bousquet (2003) characterized French-American hybrid grapevines’ genetic background using 6 microsatellite (SSR) markers and a set of 33 diagnostic RAPD markers. They found estimates of genetic diversity derived from SSRs were generally higher. Thus, additional studies are necessary in order to explain emerging patterns of distribution and genetic structure of liana plant species found in the temperate forests and to test and locate glacial refugia in eastern and southern North America.

In order to explain the diversity of phylogeographic patterns in the eastern and southern North American liana taxa, we have chosen to focus on the deciduous climbing species of the Virginia creeper genus *Parthenocissus* Planch. (Vitaceae), which are indigenous to North America. The genus shows a disjunct distribution between Asia and North America, and contains *c.* 13 species with approximately ten in eastern Asia and three in North America (Soejima and Wen, 2006; Wen, 2007; Chen et al., 2007). Based on recent molecular phylogenetic evidence (Nie et al., 2010; Lu et al., 2012; Yu et al., 2023), two main clades were recognizable within *Parthenocissus*, corresponding to their distribution in the New and the Old World (i.e., North America and Asia). In the New World, *P. vitacea* (Knerr) Hitchc. and *P. heptaphylla* (Buckl.) Britton ex Small. are morphologically similar to *P. quinquefolia* (L) Planch (Nie et al., 2010), and they have hermaphrodite flowers that produce berries dispersed by birds and mammals (Wen, 2007). This clade is an ideal model to investigate the phylogeography of post-glacial migration because *P. quinquefolia* is distributed widely across eastern and southern North America, and *P. heptaphylla* and *P. vitacea* each have a smaller distributional range, especially *P. heptaphylla*, which is only found on the Edwards Plateau in Texas (around 30°N).

In the present study, we sequenced three chloroplast regions (*rps16*, *trnL-F*, and *trnC-petN*) and one nuclear gene (*ARF6*) from all three species from North America. Based on these datasets of four gene sequences, we examine the genetic structure, phylogeographic history and mechanisms of gene flow of *Parthenocissus* in North America. We attempt to address the following questions: (i) When did diversification occur among major lineages of North American *Parthenocissus*? (ii) Where were the refugia of *Parthenocissus* in eastern and southern North America? (iii) Are there post-glacial colonization patterns in the present geographic range of *Parthenocissus* across North America?

## 2 Materials and methods

### 2.1 Sample collection and DNA extraction

We collected 398 individuals from 47 populations across North America. Our sampling extended as far north as the population in Ontario, Canada (45.1749°N and 74.8326°W), as far east as the population in Pennsylvania, USA (41.7364°N and 70.8566°W), as far south as the population in Texas, USA (29.8031°N and 98.4934°W), and as far west as the population in Texas, USA (30.7052°N and 104.2134°W), basically covering their entire distribution range from south to north of eastern North America. Three to 12 individuals per population were randomly sampled intervals were ≥ 10 m apart. After species identification, the leaflets of fresh healthy leaves were dried in silica gel and the dried leaf tissue samples were stored at -20°C for further extraction of genomic DNA. The geographic location of the populations, the number of samples and voucher information are shown in **Table 1**.

**Table 1:** Geographic and haplotype characteristics of 47 *Parthenocissus* populations from North America surveyed for chloroplast (cp) DNA sequences and nuclear ribosome (nr) DNA variation.

Pop	Voucher	Locations	Latitude (°N)	Longitude(°W)	Chloroplast haplotype frequencies	Nuclear haplotype frequencies
SA1	Wen11732	North Carolina, Pisgah	35.7145	81.7756	C1(2),C2(2),C3(3),C4(1)c	H1(4),H2(10)
SA2	Wen11751	North Carolina, Swain	35.3408	83.5742	C1(2),C3(3),C5(1),C6(2)	H1(1),H2(5),H3(2)
SA3	Wen11760	Georgia, Union	34.8233	83.9211	C2(7),C6(2),C7(1)	H1(1),H2(10),H4(2),H5(5),H6(2)
SA4	Wen11774	Georgia, Decatur	31.2917	84.8529	C2(1),C7(1),C8(4)	H2(2),H3(2),H7(2),H8(2),H9(2)
SA5	Wen11778	Florida, Liberty	30.5759	84.9487	C9(3),C10(1),C11(3),C12(1)	H2(10),H3(2),H10(2)
SA6	Wen11790	Virginia, Montgomery	37.0963	80.5623	C1(1),C3(1),C9(2),C13(1),C14(2),C15(1)	H2(4),H3(2),H5(2),H6(2)
SA7	Wen11793	Virginia, Page	38.6517	78.3543	C6(1),C8(2),C14(4)	H2(9),H5(1),H10(1),H11(2),H12(1)
SA8	Wen11968	Arkansas, Newton	36.0051	93.1857	C2(1),C11(1),C14(3),C15(5)	H2(4),H13(8),H14(2)
SA9	Wen11976	Texas, Taylor	32.2370	99.8854	C16(4),C17(1),C18(1),C19(2)	H2(2),H15(2),H16(2),H17(8)
SA10	Wen11980	Texas, Jeff Davis	30.7052	104.2134	C16(7),C19(1)	H18(14)
SA11	Wen11982	Texas, Schleicher	30.9114	100.5846	C16(1),C19(6),C20(1)	H2(2),H15(12)
SA12	Wen11985	Texas, Kimble	30.2893	99.5244	C15(1),C16(4),C17(1),C19(1),C21(1)	H2(4),H15(6)
SA13	Wen11986	Texas, Kerr	30.1949	99.3779	C22(7)	H5(14)
SA14	Wen11996	Texas, Comal	29.8031	98.4934	C2(2),C6(1),C14(1),C22(3),C23(1)	H2(4),H5(2),H8(2),H19(6)
SA15	Wen12002	Texas, Blanco	30.3626	98.2776	C16(5),C24(1),C25(2)	H1(2),H2(3),H15(11)
SA16	Wen12007	Texas, Montgomery	30.5306	95.5763	C2(1),C10(1),C14(4),C26(1)	H1(5),H2(2)H5(4),H8(1)
SA17	Wen12009	Louisiana, St. Martin Parish	30.3416	91.7202	C27(2),C28(6)	H13(16)
SA18	Wen12016	Mississippi, Scott	32.2439	89.5026	C2(1),C9(2),C14(1),C26(1),C29(2),C30(1)	H2(6)
SA19	Wen12018	Alabama, Tuscaloosa	33.1518	87.2681	C1(1),C5(1),C7(1),C9(3),C30(1),C31(1)	H3(4),H4(2),H9(2)
SA20	Wen12024	Tennessee, McMinn	35.2669	84.5443	C1(1),C9(3),C11(1),C12(1),C30(1)	H1(2),H2(8),H5(4)
SA21	Wen12194	Alabama, Baldwin	30.5217	87.8957	C2(8),C8(1)	H1(6),H5(3),H6(1),H9(3),H19(1)
SA22	Wen12199	Alabama, Mobile	30.4031	88.2481	C2(4),C7(1)	H1(6),H2(2),H3(2)
EA23	Wen12200	Virgina, Culpeper	38.5408	78.1317	C1(1),C2(10),C6(2)	H1(1),H2(9),H3(4),H6(2),H8(1),H20(2),H21(3)
EA24	Wen12203	Ohio, Richland	40.7125	82.4235	C1(4),C2(1),C3(3)	H2(10),H7(2),H11(1),H12(2),H22(1)
EA25	Wen12206	Ohio, Richland	40.6324	82.4235	C1(6),C3(4),C4(1)	H1(8),H2(14)
EA26	Wen12208	Ohio, Ashtabula	41.8772	80.7969	C1(1),C2(3),C6(2),C8(1),C19(3)	H1(4),H2(6),H4(2),H5(2),H15(4)
EA27	Wen12209	Pennsylvania, Mercer	41.2264	80.2375	C1(6),C3(1),C32(1)	H1(6),H5(9),H23(1)
EA28	Wen12211	Pennsylvania, Allegheny	40.5765	80.0293	C1(2),C19(1),C33(1),C34(2)	H5(2),H6(2),H15(4)
EA29	Wen12214	Pennsylvania, McKean	41.7364	70.8566	C3(6),C4(1)	H1(1),H2(10),H24(1)
EA30	Wen12217	Pennsylvania, Cattaraugus	42.4849	78.9506	C3(2),C4(2),C35(3)	H2(8),H15(3),H25(1)
EA41	Wen12245	Connecticut, Litchfield	41.9883	73.0471	C1(8)	H2(2),H3(1),H5(5),H7(2)
EA42	Wen12248	Connecticut, Fairfield	41.4384	73.4735	C2(7)	H2(1),H3(8),H12(1)
EA43	Wen12251	New York, Orange	41.4201	74.4258	C2(7)	H2(3),H3(3),H5(2)
EA44	Wen12252	Pennsylvania, Wayne	41.4086	75.5080	C2(10),C5(1)	H1(3),H2(5),H3(4),H5(2),H8(2),H19(2)
EA45		Michigan, East Lansing	42.7312	84.4902	C6(1),C9(2),C11(1),C14(4),C39(2),C40(1)	H1(3),H2(14),H5(2),H6(1)
NA31	Wen12219	Ontario, Grey	43.5387	80.2236	C24(7),C35(1),C36(1)	H2(2),H15(4),H26(2),H27(4)
NA32	Wen12224	Ontario, Grey	44.6140	80.7281	C35(9),C37(1)	H2(7),H15(4),H23(4),H25(1)
NA33	Wen12229	Ontario, Northumberland	44.3785	77.8674	C24(8)	H2(4),H15(2),H23(2),H26(2)
NA34	Wen12230	Ontario, Frontenac	44.7796	76.7225	C35(9),C36(2)	H1(1),H2(3),H15(6),H23(2)
NA35	Wen12231	Ontario, Glengarry	45.1749	74.8326	C6(1),C24(4),C35(1)	H2(3),H15(2),H23(3),H26(2)
NA36	Wen12233	Canada, Quebec	45.1741	73.1953	C24(8),C36(1)	H1(1),H2(1),H15(13),H23(1)
NA37	Wen12237	New Hampshire, Coos	44.6389	71.5421	C24(7)	H1(9),H7(4),H15(1)
NA38	Wen12238	Vermont, Bennington	42.8830	73.1544	C24(5),C36(1)	H1(3),H2(7),H15(2)
NA39	Wen12240	Massachusetts, Berkshire	42.3366	73.3324	C6(3),C24(8),C36(1)	H1(3),H2(12),H7(1),H15(6)
NA40	Wen12242	Massachusetts, Berkshire	42.2140	73.0979	C19(10),C36(1),C38(1)	H1(10),H2(4),H8(1),H15(1)
NA46		Michigan, Leelanau	44.2383	85.4007	C16(7),C19(1),C41(1),C42(1),C43(1),C44(1)	H1(1),H2(5),H7(7),H28(3),H29(8)
NA47		Wisconsin, West Salem	43.8969	91.0968	C16(5),C19(1),C43(1),C45(1),C46(1),C47(1)	H2(6),H15(2),H26(4),H27(6)

### 2.2 DNA extraction, gene amplification, sequencing and comparison

DNA was extracted from silica-dried leaves using a modified CTAB method (Doyle and Doyle, 1987) or using the DNeasy Plant Mini Kit (Qiagen, Crawley, UK). Amplification and sequencing followed Soejima and Wen (2006) for the plastid sequences (*trnL-F*, *rps16* and *trnC-petN*), and Ehrenreich and Purugganan (2008) for the nuclear *ARF6* gene. DNA sequences were assembled using Sequencher v4.1.4 (Gene Codes Corp., Ann Arbor, Michigan, USA). All sequences obtained were aligned using MUSCLE v3.8 (Edgar, 2004) and the alignment was then adjusted manually.

### 2.3 Genetic diversity analyses

Shared haplotypes were determined using DnaSP v5.10 (Librado and Rozas, 2009). The number of haplotypes (H) and polymorphic sites (S), haplotype diversity (H_d_), and nucleotide diversity (Pi) were calculated using DnaSP. We constructed the network relationships with cpDNA and nrDNA haplotypes using PopART v1.7 with Median-Joining model, respectively (Leigh and Bryant., 2015). Population structure and relationships among haplotypes were conducted using maximum parsimony network in PAUP v4.0 (Swofford, 2002), which was designed to construct the shortest, least complex network. In this analysis, gaps with two or more base pairs were coded as single mutation events. When overlapping indels occurred, the overlap portion was considered to be a single event.

### 2.4 Population genetic structure analysis

The software STRUCTURE v2.3.4 was used to analyze the genetic structure of the populations. Clustering method based on Bayesian model was used to assign genotypes/individuals to different clusters according to shared co-ancestry to describe the genetic structure well (Pritchard et al., 2000). The software was run using the Admixture Model with parameters set to 20,000 burn-in repeats and 70,000 MCMC repeats. The number of clusters (K) was set to vary from two to 12. For each value of K, we performed was 20 runs. The relationship between K and LNP (D) and ΔK calculated by Structure Harvester was used to obtain the best K value.

An analysis of molecular variance (AMOVA) was used to partition genetic variation among and within groups, as implemented in ARLEQUIN v3.5 (Excoffier and Lischer, 2010). G_ST_ and N_ST_ among populations were calculated from the chloroplast markers using 1000 permutations in PermutCpSSR v2.0 (Burban et al., 2010). The principal coordinate analysis (PCoA, Peakall and Smouse, 2006) was carried out using DARwin v7.0 software (Perrier and Jacquemoud-Collet, 2006). Nei’s genetic distance among the populations of *Parthenocissus* was calculated in MEGA v7.0, and Neighbor-Joining (NJ) trees were constructed for 47 population using the Nei’s genetic distance (Kumar et al., 2016; Nei, 1972).

### 2.5 Population history dynamic analysis and divergence time estimate

We examined pairwise mismatch distributions based on the pairwise nucleotide differences between haplotypes to detect demographic expansions using ARLEQUIN. Populations at demographic equilibrium should present a multimodal or random and rough distribution of pairwise differences, whereas populations experiencing a sudden demographic expansion are expected to display a unimodal and smooth distribution (Slatkin and Hudson, 1991). In order to test whether the overall distribution area and different populations of *Parthenocissus* had expanded historically, we conducted neutrality tests by DnaSP based on cpDNA and nrDNA.

The divergence time estimate was conducted in BEAST 1.8.4 (Drummond and Rambaut, 2007) using the dataset including 47 populations with *Parthenocissus chinensis* as outgroup. The dating dataset was partitioned using BEAUti 1.8.4 to generate input files for BEAST. Under the Akaike information criterion (AIC) implemented in MrModeltest 2.3, the best-fit model of nucleotide substitution for this analysis was determined to be HKY (Posada and Crandall, 1998). We applied the HKY model under an uncorrelated lognormal relaxed clock model (Drummond et al., 2006). MCMC analyses of 100,000,000 generations were implemented, in which every 1,000 generations were sampled. The first 10% of generations were discarded as burn-in, and the parameters were checked using the program Tracer 1.6, when the effective sample size of all parameters exceeds 200, the results were considered reliable. The rest sampled posterior trees were summarized to generate a maximum clade credibility tree using the program TreeAnnotator 1.8.4 (Drummond and Rambaut, 2007). The program Figuretree 1.4 (Drummond and Rambaut, 2007) was used to compile and visualize the results from BEAST. According to Rogers and Harpending (1992), the formula T=τ/2μkg was applied to calculate the population expansion time (τ: expansion parameter from mismatch distribution analysis; μ: mutation rate; k: average sequence length of the cpDNA region under study, the value is 1223bp, see the Results section; g: Generation time of *Parthenocissus*, calculated in 3 years, Li, 1998).

## 3 Results

### 3.1 Genetic diversity

The total alignment of the three chloroplast regions (*trnL-F*, *rps16* and *trnC-petN*) surveyed across all the individuals was 1223 bp, and 38 polymorphic sites were observed, all of which were indels. A total of 47 chloroplast haplotypes (C1-C47) were identified (**Table 1**). The nuclear *ARF6* gene was 488 bp long with 16 polymorphic sites, including 1 indel and 15 base substitutions. We identified 29 nuclear haplotypes (H1-29) across the 47 surveyed populations (**Table 1**).At the species level, the cpDNA data showed a higher estimates of haplotype diversity (Hd = 0.9276) than the value from the nrDNA data (Hd = 0.8331). However, the nucleotide diversity of nrDNA (pi= 4.06×10^-3^;) was higher than that of cpDNA (pi = 3.99×^-3^) (**Table 2**). The C2 and H2 haplotypes were most common in *Parthenocissus*, with a frequency of 16.3% (65 accessions and 15 populations) and 68.2% (223 accessions and 39 populations), respectively. The distribution frequency of C2 and H2 was high (**Table 1**), basically located in the center of their branches.

**Table 2:** Estimates of average gene diversity within populations (H_S_), total gene diversity (H_T_), interpopulation differentiation (G_ST_), number of substitution types (N_ST_) and haplotype diversity (H_d_) within *Parthenocissus*.

Regions	H_d_	H_S_	H_T_	G_ST_	N_ST_	Tajima’s D	SSD	*H_Rag_*
cpDNA								
Total	0.9276	0.519	0.940	0.444*	0.488*	6.54052*	0.13253*	0.16635
SA	0.9320	0.637	0.947	0.328	0.290	6.57882*	0.11033*	0.14682
EA	0.7482	0.385	0.816	0.528	0.561	2.56713*	0.056533	0.12077
NA	0.7296	0.436	0.814	0.464	0.533	0.98599	–	–
nrDNA								
Total	0.8331	0.585	0.844	0.306*	0.457*	-0.15663	0.00258	0.02368
SA	0.8540	0.532	0.855	0.377*	0.524*	0. 53297	0.00230	0.12613
EA	0.7640	0.613	0.796	0.230*	0.291*	0.47430	0.00369	0.03419
NA	0.7582	0.672	0.817	0.177*	0.266*	0.35804	–	–

Neutrality tests and mismatch distribution analysis for different regions of cpDNA and nrDNA for *Parthenocissus* from North America.

* Significant at *P*<0.05.

**Figure 1:**
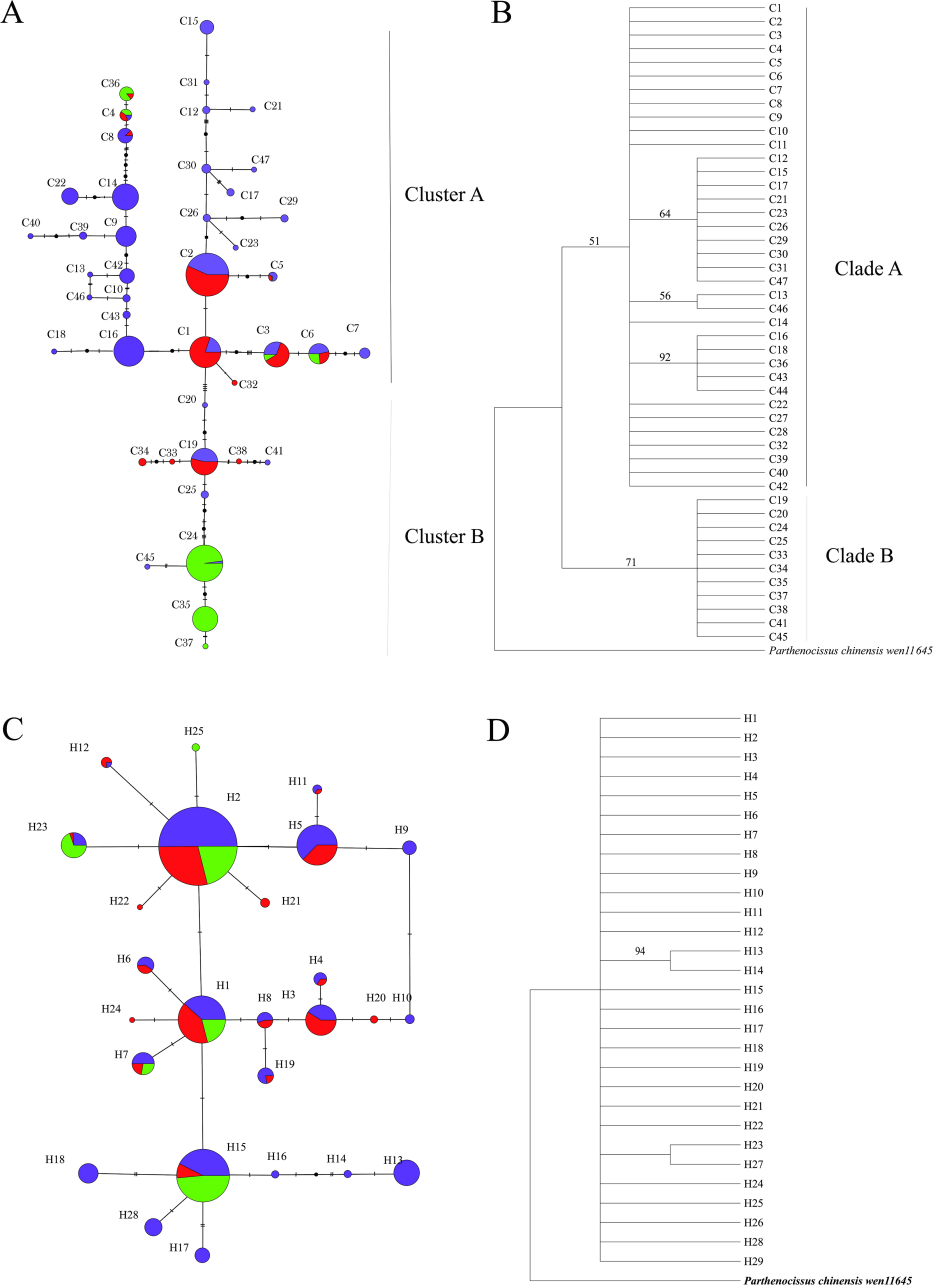
TCS-derived network of genealogical relationships in popART between the 47 haplotypes from cpDNA **(A)** and 29 haplotypes from nrDNA **(C)**. Stric consensus of parsimonious trees for 47 chlorotypes **(B)** and 29 nuclear haplotypes **(D)** of *Parthenocissus*, respectively. Each circle means a single haplotype sized in proportion to its frequency. Small black circles represent missing haplotypes. Numbers on the branches indicate the bootstrap values for maximum parsimony analyses.

The parsimony network grouped the 47 cpDNA haplotypes into two major clusters (Cluster A and Cluster B) separated by 4 mutational steps (**Figure 1A**). Thus, each region mostly harbored a genealogically distinct set of haplotypes. The cluster A included 32 cpDNA haplotypes. These cpDNA haplotypes were found quite broadly from the Southern North America region (SA, 23 unique haplotypes and 7 shared haplotypes). Note that C1, C2 and C16 were central haplotypes from which other haplotypes diverged. There were 11 haplotypes in cluster B diverged from center on C19 and C24. Meanwhile, a haplotype network was constructed based on 29 nrDNA haplotypes (**Figure 1C**). The plausible network tree of nrDNA had three clusters centering on H1, H2 and H15, and other haplotypes diverged from these three centers. The phylogenetic trees resulting from MP of cpDNA and nrDNA (**Figures 1B, D**) supported a similar pattern observed in the network analysis.

### 3.2 Population genetic structure

**Figure 2:**
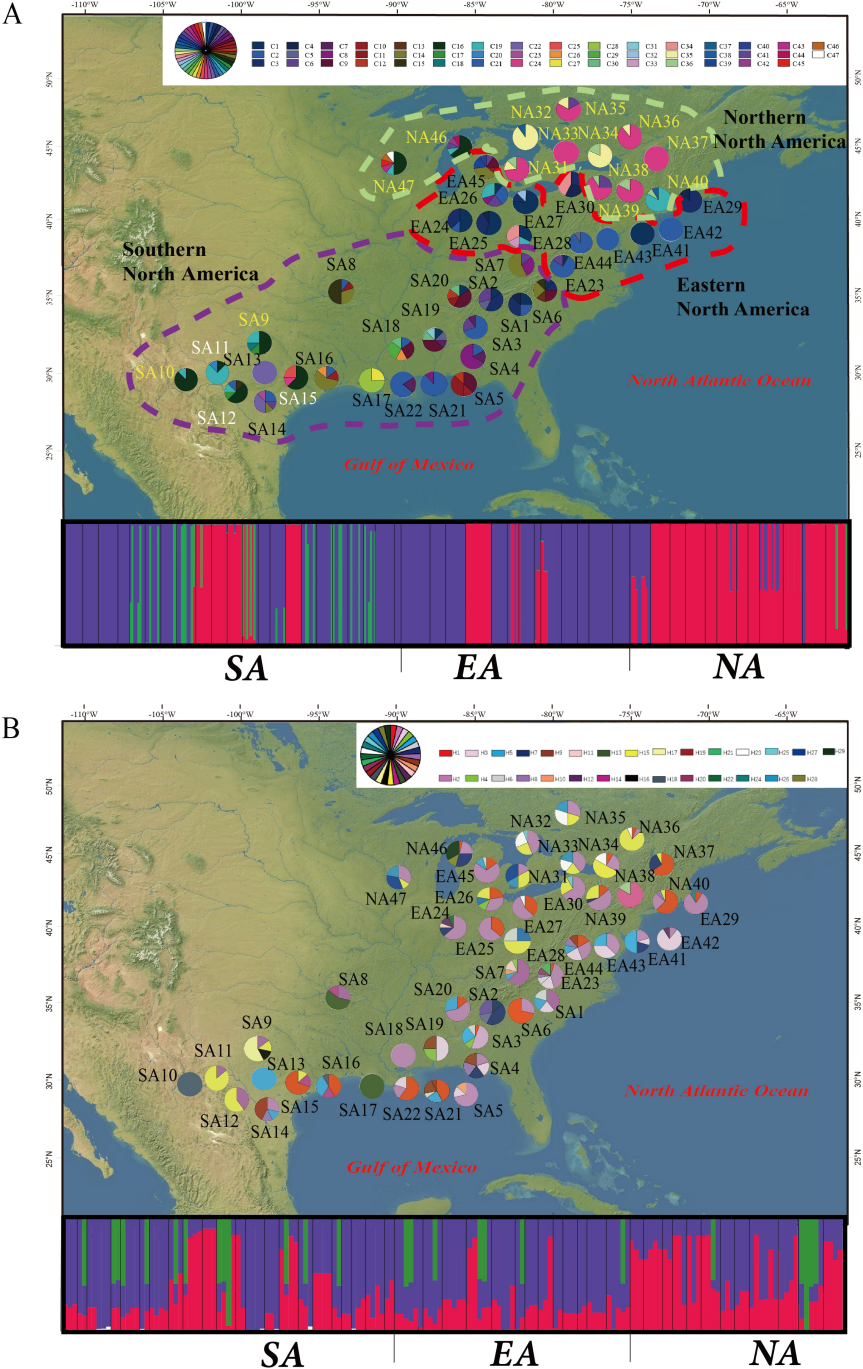
Geographic distribution of 47 cpDNA haplotypes **(A)** and 29 nrDNA haplotypes **(B)** detected in 47 populations of *Parthenocissus* from North America. The dashed circles delimitate the three population groups detected by STRUCTURE analysis, comprising three large groups including Southern North America region (SA, purple dashed line), Eastern North America region (EA, red dashed line) and Northern North America region (NA, green dashed line). The black text represents *P. quinquefolia*, the yellow text represents *P. heptaphylla*, and the white text represents *P. heptaphylla*. Histogram of the STRUCTURE analysis for the model with K = 3, the smallest vertical bar represents one individual. The assignment proportion of each individual into one of four population clusters is shown along the y-axis.

In the case of the STRUCTURE analysis, we detected three phylogeographic groups (F_CT_ = 0.48124, p<0.001) as the optimal number of genetic “groups” (K) based on spatial locations and cpDNA haplotypes (**Table 3**, **Figure 2A**). Interestingly, these phenomena appeared in nrDNA but were not very obvious (**Figure 2B**). The southern North American (SA) group was the largest grouping with 22 populations (SA1-22), which were distributed across Texas, Arkansas, Louisiana, Mississippi, Alabama, Tennessee, Georgia, North Carolina, Florida and Virginia. The eastern North American (EA) group included 13 populations from Virginia, Connecticut, New York and Pennsylvania (EA23-30 and EA41-45). The northern North American (NA) group was exclusively located in Ontario, Quebec and Massachusetts (NA31-40 and NA46-47), and this assemblage contained 12 populations (**Figure 2**). We also conducted haplotypes and STRUCTURE analyses for *P. quinquefolia* and *P. vitacea* - *P. heptaphylla* separately (**Supplementary Figures S1**, **S2**). The results showed that no clear biogeographic pattern was found in *P. quinquefolia*, but *P. heptaphylla* and *P. vitacea* could be divided into two groups (**Supplementary Figure S2**), the southern group mainly occurred on the Edwards Plateau in Central Texas, and the northern group consisted of *P. vitacea* in Canada and the northern USA (**Supplementary Figure S1**, **Supplementary Table S1**).

**Table 3:** The analysis of molecular variance (AMOVA) for cpDNA data and nrDNA data among three geographic regions (Southern North America, Eastern North America, Northern North America) and all populations of *Parthenocissus* from North America.

source of variation	cpDNA					nrDNA				
	df	sum of squares	variance components	percentage of variation (%)	fixation indices	df	sum of squares	variance components	percentage of variation (%)	fixation indices
Three geographic groups										
Among populations	1	399.080	1.46621	29.40	F_SC_=0.04619*	1	24.173	0.06281	5.96	F_SC_=0.03273*
Among populations within groups	1	23.613	0.07416	1.49	F_ST_=0.50015*	1	10.157	0.02087	1.98	F_ST_=0.0830*
Within populations	395	1115.61	3.44713	69.11	F_CT_=0.48124*	651	638.415	0.97063	92.06	F_CT_=0.06385*
Total population										
Among populations	2	378.189	1.74042	33.55	F_ST_=0.33550*	2	34.327	0.07636	7.22	F_ST_=0.07224*
Within populations	395	1115.61	3.44713	66.45		651	638.415	0.98067	92.78	

* Significant at *P*<0.001

**Figure 3:**
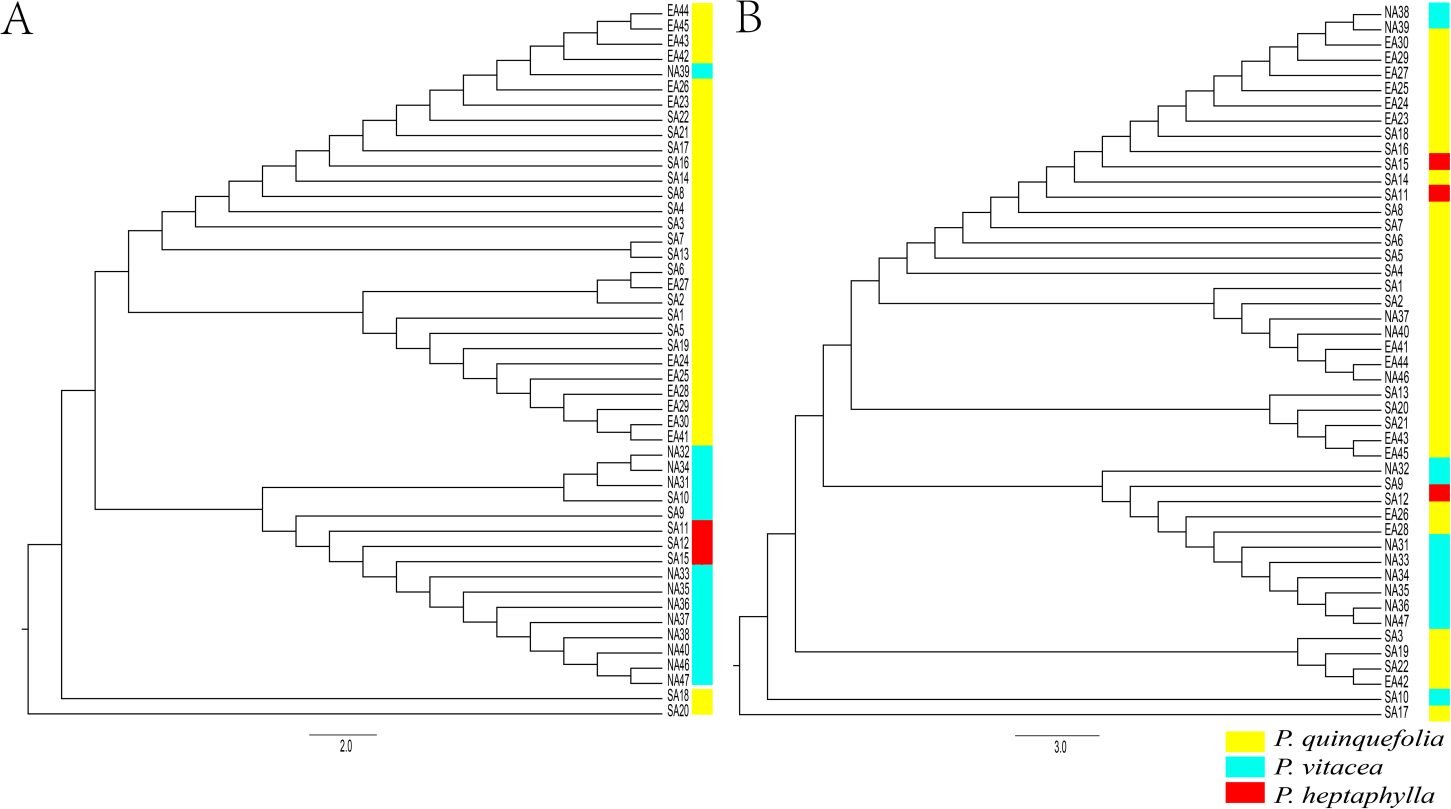
The UPGMA dendrogram based on Nei’s (1972) genetic distance among 47 populations of *Parthenocissus* inferred from cpDNA **(A)** and nrDNA **(B)** sequences.

A permutation test showed that N_ST =_ 0.488 was significantly greater than G_ST_ (0. 0.444, P < 0.05) in cpDNA, and that N_ST =_ 0.457 was significantly greater than G_ST_ (0. 0.306, P < 0.05) in nrDNA (**Table 2**). In terms of AMOVA results based on cpDNA, approximately 66.45% of total variation was explained by differences within populations and 33.55% due to differences among populations. For nrDNA, 92.78% of variation was partitioned within populations and 7.22% among populations (**Table 3**). For the three recognized groups in STRUCTURE, approximately 69.11% of variations occurred within populations, and the remaining 29.40% and 1.49% occurred among populations and among populations within groups in cpDNA, respectively. Meanwhile, 92.06% of variation was partitioned within populations, and the remaining 5.96% and 1.98% among populations and among populations within groups in nrDNA, respectively (**Table 3**).

**Figure 4:**
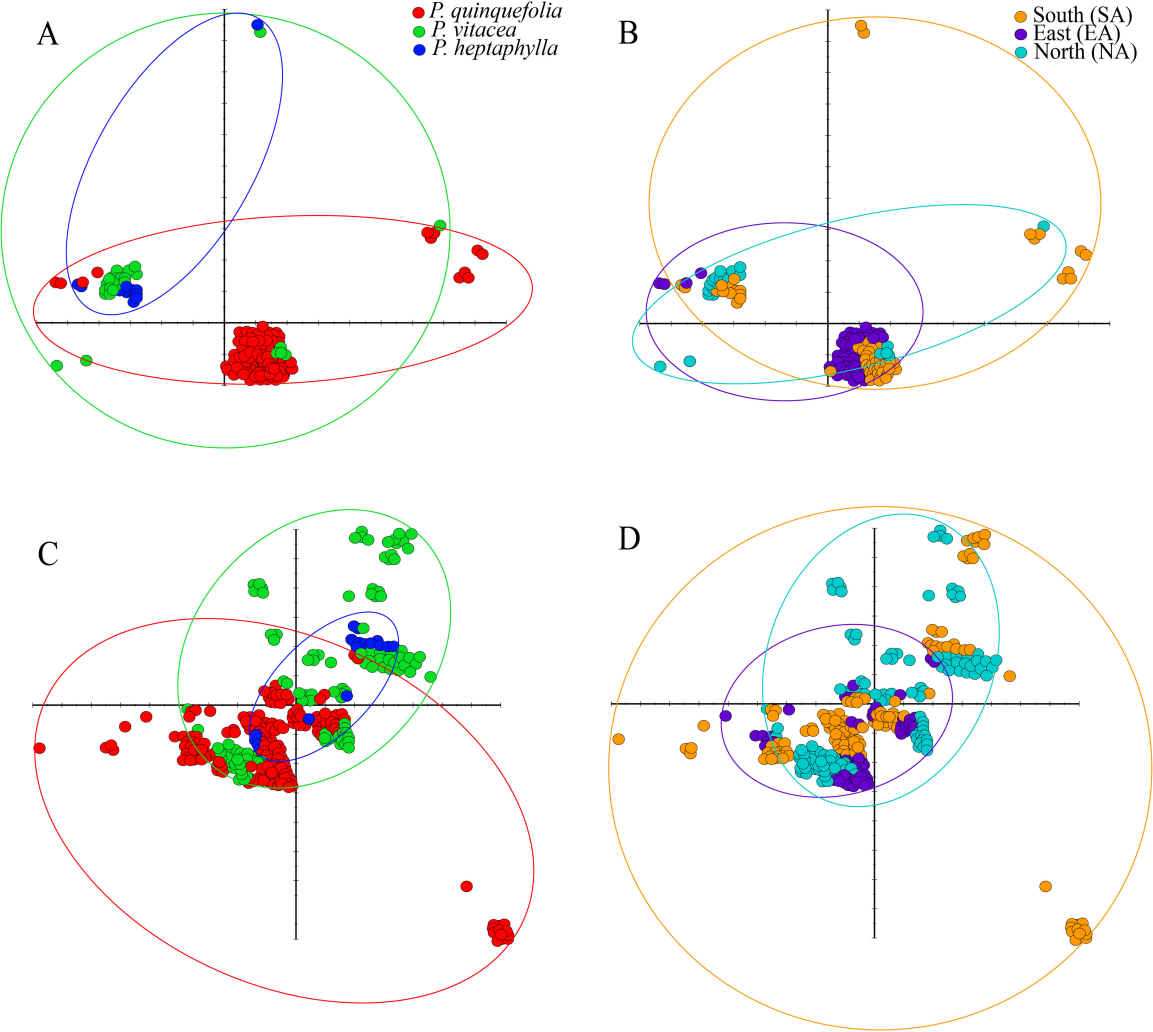
Principal coordinate analysis (PCoA) for 47 populations of *Parthenocissus* inferred from cpDNA **(A, B)** and nrDNA **(C, D)** sequences.

The UPGMA clustering tree constructed based on Nei’s genetic distance indicated that nrDNA showed more complex pattern than cpDNA (**Figure 3**). Principal coordinate analysis based on similarity matrix also agreed with UPGMA structure and STRUTURE results (**Figure 4**; **Supplementary Figure S2**).

### 3.3 Population history dynamic and estimations of divergence times

**Figure 5:**
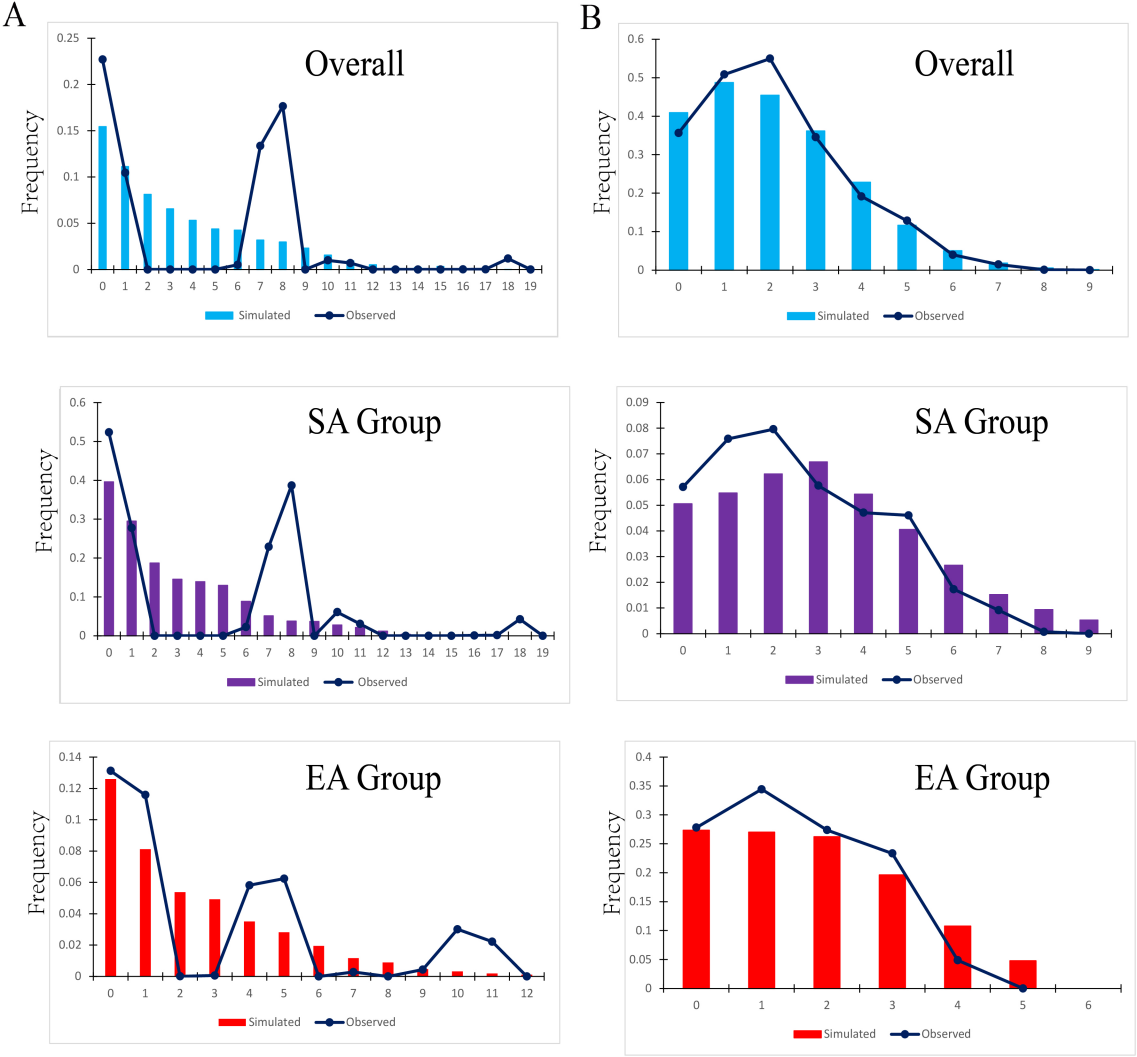
Historical demography of *Parthenocissus* inferred from cpDNA and nrDNA sequences. **(A)** Pairwise mismatch distributions for cpDNA clades. **(B)** Pairwise mismatch distributions for nrDNA clades.

Our cpDNA results showed that there are no explicit signals of population expansion or equilibrium in neutrality tests. The observed mismatch distribution of EA regions did not reject the spatial expansion model (**Table 2**), but a unimodal distribution was not identified in all regions (**Figure 5A**). Estimates of Tajima’s were generally nonsignificant for all nrDNA regions of *Parthenocissus* (**Table 2**). By contrast, the mismatch distribution in nrDNA showed that the overall population was unimodal (**Figure 5B**), closely fitted to the expected distribution under the sudden expansion model. The Sum of Squared deviation (SSD) of 0.00258 (p>0.05) and Harpending’s Raggedness index (*H_Rag_*) of 0.02368 (p>0.05; **Table 2**) could not reject the population expansion model.

**Figure 6:**
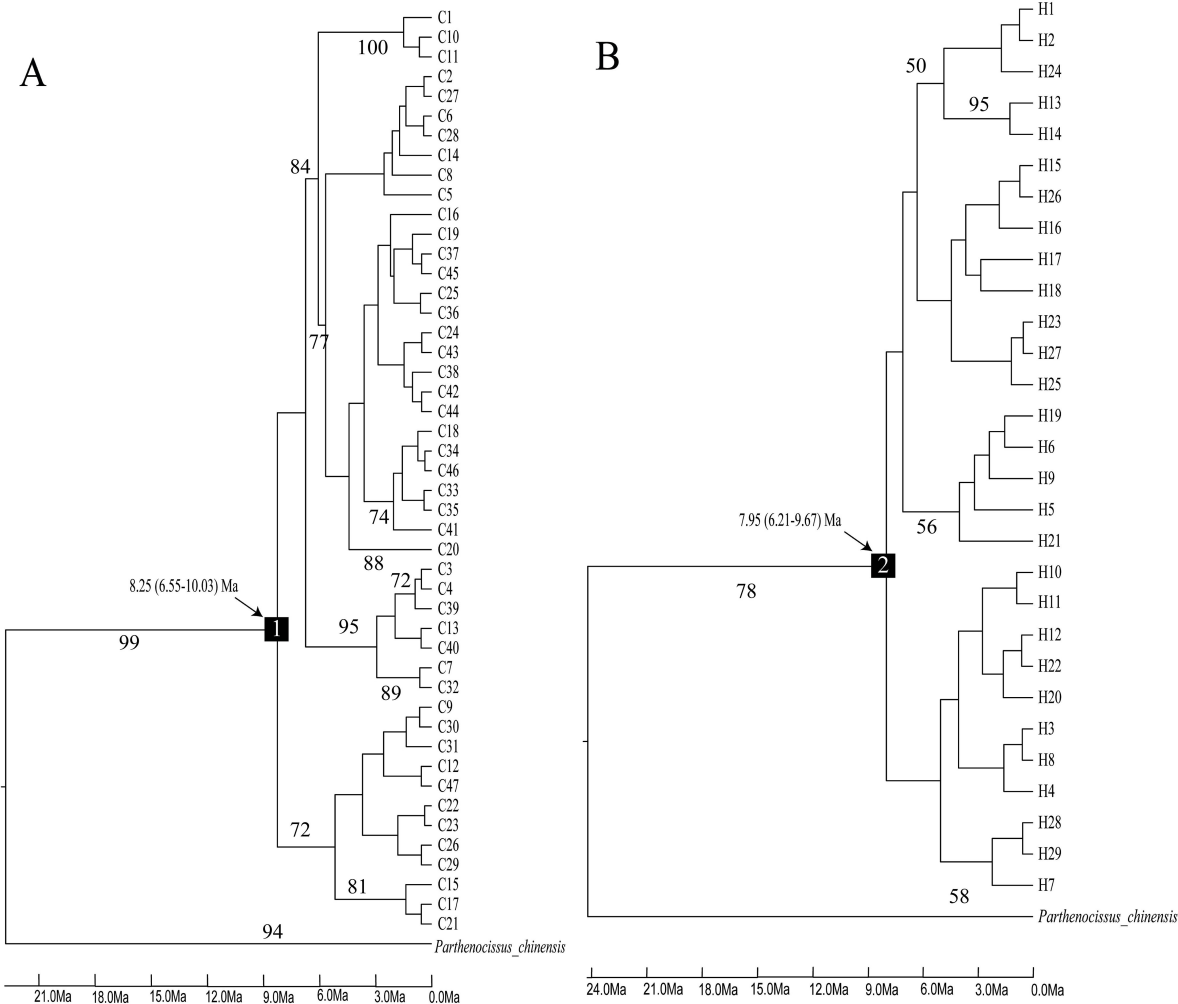
BEAST-derived chronograms about Parthenocissus populations based on cpDNA **(A)** and nrDNA **(B)** sequences. The numbers (1-2) represent the mean divergence age of the North American group. Haplotypes are indicated by letter codes (C1-47; H 1-29).

The BEAST analyses based on two calibration points suggested an origin of the North American *Parthenocissus* crown lineage at 8.25 Ma with a 95% HPD of 6.55-10.03 Ma based on the combined cpDNA (**Figure 6A**). The crown node age of *Parthenocissus* was estimated to be 7.95 Ma with a 95% HPD of 6.21-9.67 Ma in nrDNA data (**Figure 6B**). The expansion time was estimated to be 0.074-0.604 Ma.

## 4 Discussion

### 4.1 High genetic diversity in North America

Our results of cpDNA and nrDNA haplotypes demonstrate a high level of genetic diversity across the 47 populations of *Parthenocissus* in North America (**Table 2**). A possible explanation for the high diversity in these species could be its long evolutionary history, which may have allowed accumulation of genetic variation. There are some characteristics in *Parthenocissus* such as hermaphroditism, attachment to various trees, and fruits that attract birds for seed spread (Wen, 2007; Moran et al., 2009; Tiffney and Barghoorn, 1976), which have led these species to adapt and evolve under diverse habitats. Williams et al. (2004) utilized a fossil-based data set from over 700 sites in northern and eastern North America to review the late-Quaternary vegetation history of this region at different ecological organizational levels, from individual taxa to biomes. They found that during the full-glacial period (21,000 - 17,000 yr ago [calendar years]) and the mid- to late-Holocene (7,000 - 500 yr ago), the distribution and composition of the vegetation were relatively stable. However, rapid changes occurred during the late glacial- and early-Holocene (16,000 - 8,000 yr ago) and after 500 yr ago. Besides the northward redistribution of most taxa, large - scale east - west distribution shifts were also observed. The wide geographic ranges of those species across North America have provided ample opportunity for isolation, drift and mutation (Wang et al., 2009). This finding is not surprising, as there are many species with high genetic diversity in North America, such as *Trillium grandiflorum*, *Smilax hispida*, *Smilax rotundifolia* and *Picea glauca.* We found that they have similar dispersal mechanisms. For example, *T. grandiflorum* spreads its pollen through bumblebees and its seeds through white-tailed deer (Griffin and Barrett, 2004). Smilax has small fleshy fruits, and its seeds are dispersed by birds while its pollen is spread by insects (Zhao et al., 2013). Additionally, *Picea glauca* has its seeds dispersed by birds and its pollen dispersed by the wind (O’Connell et al., 2007). Moreover, Nadeau et al. (2015) suggested that high levels of genetic diversity were maintained across the range of *Pinus strobus*, likely via frequent long-distance dispersal events during colonization. O’Connell et al. (2007) also pointed out in the study on white spruce that extensive long-distance, pollen-mediated gene flow seems to be the primary mechanism for maintaining genetic diversity among the populations.

### 4.2 Extensive gene flow

EI-Kassaby (1991) and Hamrick et al. (1992) conducted phylogeographic studies of forest woody plants and found that partitioning of the genetic variability often reveals that more than 90% of the total genetic variation resides within populations and less than 10% is due to differentiation among populations. In these cases, gene flow was thought to be the main forces shaping the population genetic structure of each species (Wang and Szmidt, 2001). Some accessions of the three species show evidence of admixture (**Figure 2**), which might be attributable to recent introgression events. Our results suggest that 66.45% of cpDNA and 92.78% of nrDNA genetic variation existed within populations, and significant genetic differentiation (**Table 3**), which indicates that a wide range of gene flow have occurred among the *Parthenocissus* populations in North America. This result raises the question of what mechanisms might account for the gene flow among populations of *Parthenocissus*.

A possible explanation is that cpDNA represents maternal inheritance reflecting the dispersal path and distance of seeds, and nrDNA represents biparental inheritance that depends on both seed and pollen transmission (Schaal et al., 1998). In *Parthenocissus*, the seeds can be spread via ingestion and defecation by birds (like *Cyanopica cyanus*), increasing gene flow between populations (Worth et al., 2010; Schaefer et al., 2009). The long-distance seed dispersal could contribute to post-glacial recolonization (Worth et al., 2010). At the same time, an alternative mechanism of gene flow between populations has been suggested by Griffin and Barrett (2004) that bumble bees could mediate pollination between populations as the predominant pollinators of *Trillium grandiflorum*, and he found that pollen flow between populations was more likely than seed propagation. Our findings may support this hypothesis. Some insects can also play the same role of bumble bees for *Parthenocissus* in eastern North America, like Syrphidae and *Apis mellifera ligustica* (Robertson, 1984). We have also found that *Parthenocissus* plants have the characteristics of both anemophily and entomophily. Pollination via insects and wind and bird-mediated seed dispersal are the primary agents of gene flow between populations of *Parthenocissus* in eastern and southern North American (Robertson, 1984; Johnson and Hendrix, 2010; Thompson and Kevan, 2012). Therefore, the cpDNA shows that the gene flow among populations is greater than that within populations, while the nrDNA is based on the seed flow plus the pollen flow, and the gene flow between population is more extensive. Based on this, we believe that pollen and seed are predominantly wind dispersed.

Genetic diversity and Permut analyses indicate that cpDNA haplotypes show higher genetic diversity and more obvious phylogeographic structure than nrDNA (**Table 2**). This result is also confirmed by AMOVA and PCA results (**Table 3**; **Figure 4**) that cpDNA data show greater genetic differentiation than nrDNA data (cpDNA: F_ST=_0.33550, nrDNA: F_ST=_0.07224; **Table 3**). Similarly, this is likely due to seed-mediated maternal inheritance of chloroplast genome in *Parthenocissus*, while biparental inheritance in nuclear genome is dependent on both seeds and pollen mediation. In the population history, cpDNA gene flow by seeds is limited, while nrDNA has lost some phylogeographic structure through extensive wind-mediated pollen flow (Zinck and Rajora, 2016; Wang and Szmidt, 2001). Zinck and Rajora (2016) used the chloroplast microsatellite and nuclear markers data to show that in conifers, the chloroplast genome is paternally inherited through pollen-mediated processes. Compared with seed-mediated gene spread, pollen-mediated long-distance gene dispersal is more common.

### 4.3 Refugia in southern part of eastern North America

Our cpDNA results show that *Parthenocissus* consists of three main lineages, corresponding to three distinct geographic ranges of SA, EA and NA in North America (**Figures 2**, **3**). The SA has the highest genetic diversity, consistent with their high morphological variation in this region. Within North America, the seven-leaflet character state in *P. heptaphylla* was inferred to have arisen from the five-leaflet (*P. quinquefolia* and *P. vitacea*) (Nie et al., 2010; Yu et al., 2023). Our findings are more inclined to support the view that *P. vitacea* gave rise to *P. heptaphylla* in North America (**Supplementary Figure S1**). *Parthenocissus heptaphylla* is distributed in the southern region, and this morphological derivation is also in line with the characteristics of high genetic diversity in the region. Our UPGMA results also support the *P. quinquefolia* populations from SA group are the dominant taxa in the eastern and southern North American population of *Parthenocissus*, possessing the largest number of haplotypes and *P. heptaphylla* and *P. vitacea* are more closely related (Lu et al., 2012).

Phylogeographic studies have shown that the genetic diversity and genetic differentiation of glacial refugia are usually higher than in non-refuge areas of the same period, because glacial shelters usually have a stable ecological environment sufficient to withstand adverse environmental factors, allowing species to survive in the region while accumulating rich genetic diversity (Tzedakis et al., 2002; Mohn et al., 2021). There are many examples of phylogeographic studies in southeastern North America, and all the results show that this region generally exhibits extremely high genetic diversity, such as *Quercus alba*, *Acer rubrum*, *Tsuga canadensis*, *Cornus florida* (Avise, 2000). Our results indicate that most populations in SA are located in diverse habitats in the southern Appalachian Mountains, coastal forests of the Gulf of Mexico, and forests of the Edwards Plateau, where we found the largest number of shared haplotypes and ancient haplotypes, and the highest genetic diversity (**Figure 1**; **Tables 1**, **2**). It seems that *Parthenocissus* is able to maintain excellent gene exchange and variation over the course of its long-term development. Typically, this implies that *Parthenocissus* possesses strong adaptability and survival capabilities. Moreover, this region is even more likely to serve as a haven for its long-term stable survival and diffusion. Especially for the Edwards Plateau, we find that our samples of three species taken from Texas also lie within the Edwards Plateau, the eastern periphery of which is typically known as Texas Hill Country. The Edwards Plateau is a crucial ecological region, featuring distinctive topography and unique vegetation (Fowler and Dunlap, 1986). It is worth mentioning that the biogeographic analyses of the animals existing in the region like mammals, birds and reptiles also have suggested that the Edwards Plateau may have acted as a transition zone or barrier for terrestrial vertebrate dispersals (Blair, 1950; Gehlbach, 1991). The relatively complex terrains and higher temperature conditions in southern North America are conducive to the preservation and development of plant populations, resulting in high genetic diversity in the region (Sewell et al., 1996).

It is notable that the haplotype C13 within the SA group is restricted from the eastern side of the Appalachian Mountains (SA1, SA2, SA6, SA7), which probably indicated a past fragmentation into two refugia on either side of the Appalachians during the Wisconsin glaciation and these mountains may have restricted this haplotype from dispersing from its refugia. Therefore, most of the recolonization of glaciated regions (through long-distance dispersal) may have occurred from refugia south of the Appalachian Mountains (Taberlet et al., 1998; Hewitt, 2000). However, haplotype C14 is found in five populations in Arkansas, Tennessee, Mississippi, and Michigan (**Figure 2**), which likely suggests that there has been some secondary contact between the putative refugia. Similar phenomenon is also found in the nrDNA haplotype data, which implies that the barrier imposed by the Appalachians may not have been an absolute one and there is extensive hybridization and gene flow between populations, especially between *P. quinquefolia* and *P. vitacea* (Griffin and Barrett, 2004; Kim et al., 2018).

Most interesting is that the population genetic diversity of the northeastern North America is also relatively high, with the preservation of some older haplotypes (i.e., C2 and H2; **Table 1**), indicating that *Parthenocissus* may have survived in a later refugium in eastern North America, possibly near the Atlantic coastal plain (Soltis et al., 2006). Previous studies on *Ambystoma tigrinum*, *Liquidambar styraciflua* and *Pinus monticola* found evidence for two independent refugia along the Atlantic coastal plain, one of which is centered on the Carolina coast, which is consistent with what we have found here (Church et al., 2003; Morris et al., 2008; Nadeau et al., 2015). Further we found that the *Parthenocissus* species in this refuge have contributed much less to recolonization than in the SA region, although additional data are needed to assess this hypothesis.

### 4.4 Divergence in eastern North America with south to north expansion

The North American *Parthenocissus* diverged at 7.9-8.25 Ma (**Figure 6**), and underwent past range expansion around 0.074-0.604 Ma (**Table 2**, **Figures 1**, **6**). The early differentiation of *Parthenocissus* in North America can be traced back to the late Miocene probably triggered by alternating cold and warm and cold dry climates and different geographic environments caused by the strengthening of winter monsoons in the late Miocene and Pliocene. When the climate gradually became dry and cold, the temperate deciduous broadleaved forest expanded (Zachos et al., 2001). We presume that the ancestral population of *Parthenocissus* proliferated in the deciduous broadleaved forest, and the distinct microclimates in different geographic environments led to the formation of *P. quinquefolia*, *P. heptaphylla* and *P. vitacea*.

The most recent glaciation ended in North America about 10,000 years ago (Prentice et al., 1991). As the climate warmed and the glaciers retreated, eastern and western taxa began to move through the mountains, particularly along low-elevation channels that would serve as conduits for collisions between previously isolated taxa (Hewitt, 1996, 1999; Bemmels and Dick, 2018). In eastern and southern North America, the south-north orientation of the Appalachians Mountains and the Edwards Plateau allowed a large number of possible recolonization routes, where the main refugia have been inferred (Soltis and Kuzoff, 1995, 2006; Swenson and Howard, 2005; Barrow et al., 2015; Péros et al., 2021) and the vegetation dynamics after LGM were abundantly documented (Davis, 2001; Li et al., 2013; Ma et al., 2021; Peterson and Graves, 2016).

An important characteristic of *Parthenocissus* is that genetic diversity decreases along the latitudes, showing a phenomenon of decreasing from south to north (highest in the SA group; see **Table 2**). As demonstrated by many phylogeographic studies, when the ice retreated, these high latitude northern areas were rapidly colonized from the south, resulting in lower genetic diversity in the NA populations in northern North America. The loss of genetic diversity has been clearly demonstrated in a range of species, including many fish species (Hebert et al., 2011). In the Pacific Northwest of America, numerous consistent studies have been conducted on plants and animals that expanded northwestward from refugium south of the Cordilleran ice sheet, and these studies have shown a decrease in genetic diversity with the expansion (Soltis et al., 1997; Conroy and Cook, 2000). This south-north pattern of genetic diversity is consistent with the possible repeated founder effect during northward post-glacial migration from a southern Pleistocene refugium.

## 5 Conclusions

By analyzing both cpDNA and nrDNA data in a phylogeographic framework, we find that *Parthenocissus* in eastern and southern North America exhibits high genetic diversity and extensive gene flow. Our results demonstrate that populations of *Parthenocissus* in North America can be roughly separated into three main lineages and they display obvious phylogeographic structure, which may have been isolated and diverged due to climatic and geographic environmental influences since the late Miocene. This study also reveals that the Edwards Plateau, the southern Appalachian Mountains and the Atlantic coastal plains are likely glacial refugia for the *Parthenocissus* species in eastern North America. During the Pleistocene, gene introgression occurred during migration from south to north in the Appalachia Mountains and the Edwards Plateau due to incomplete reproductive isolation between sympatric species, resulting in extensive gene flow and interspecific hybridization events. Our large samples of the clade of three North American *Parthenocissus* species allowed us to provide the first reliable estimates of their genetic diversity, and genetic structure. However, the nuclear data from this study are still preliminary and insufficient. Therefore, higher density geographic sampling and more comprehensive genome-wide data will help further assess the genetic diversity and phylogeographic history. Such studies will undoubtedly lead to a better understanding of the biogeographic history of the wide-ranging plant community that characterizes the rich forested regions of eastern and southern North America.

## Data Availability

DNA sequences were deposited in the GenBank for rps16 (PV582503-PV582742), trnC-petN (PV582743-PV582983), trnL-F (PV790606-PV790965), and AFR6 (PV665093-PV665419).

## References

[B1] Avise J. C. (2000). Phylogeography: the history and formation of species (Cambridge, Massachusetts, USA: Harvard University Press).

[B2] Barnard-Kubow K. B. Debban C. L. Galloway L. F. (2015 1842). Multiple glacial refugia lead to genetic structuring and the potential for reproductive isolation in a herbaceous plant. Am. J. Botany 102 (11), 1842. doi: 10.3732/ajb.1500267 26542847

[B3] Barrow L. N. Bigelow A. T. Phillips C. A. Lemmon E. M. (2015). Phylogeographic inference using Bayesian model comparison across a fragmented chorus frog species complex. Mol. Ecology 24, 4739–4758. doi: 10.1111/mec.13343 26270246

[B4] Bemmels J. B. Dick C. W. (2018). Genomic evidence of a widespread southern distribution during the Last Glacial Maximum for two eastern North American hickory species. J. Biogeography 45 (8), 1739–1750. doi: 10.1111/jbi.13358

[B5] Blair W. F. (1950). Biotic provinces of texas. Texas J. Science 2, 93–116.

[B6] Burban C. Petit R. J. Carcreff E. Jactel H. (2010). Rangewide variation of the maritime pine bast scale Matsucoccus feytaudi Duc. (Homoptera: Matsucoccidae) in relation to the genetic structure of its host. Mol. Ecol. 8 (10), 1593–1602. doi: 10.1046/j.1365-294x.1999.00739.x 10583823

[B7] Chen Z. D. Ren H. Wen J. (2007). “Vitaceae,” in Flora of China, vol. 12 . Eds. Wu Z. Y. Hong D. Y. Raven P. H. (Science Press; and St Louis: Missouri Botanical Garden Press, Beijing), 173–177.

[B8] Church S. A. Kraus J. M. Mitchell J. C. Church D. R. Taylor D. R. (2003). Evidence for multiple Pleistocene refugia in the postglacial expansion of the eastern tiger salamander, Ambystoma tigrinum tigrinum. Evolution 57, 372–383. doi: 10.1111/j.0014-3820.2003.tb00271.x 12683533

[B9] Conroy C. J. Cook J. A. (2000). Phylogeography of a post-glacial colonizer: Microtus longicaudus (Rodentia: Muridae). Mol. Ecology 9, 165–175. doi: 10.1046/j.1365-294x.2000.00846.x 10672160

[B10] Cox C. B. Healey I. N. Moore P. D. (1977). Biogeography: an ecological and evolutionary approach. Systematic Bot 2 (3), 208. doi: 10.2307/2418264

[B11] Critchfield W. B. (1984). Impact of the Pleistocene on the genetic structure of North American conifers. Presented at 8th North Am. For. Biol. (Logan: Workshop, Utah State Univ.), 70–118.

[B12] Culver D. C. Master L. L. Christman M. C. Hobbs H. H. III. (2000). Obligate cave fauna of the 48 contiguous United States. Conserv. Biol 14, 386–401. doi: 10.1046/j.1523-1739.2000.99026.x

[B13] Davis M. B. (1983). Quaternary history of deciduous forests of eastern north america and europe. Ann. Missouri Botanical Garden 70, 550–563. doi: 10.2307/2992086

[B14] Davis M. B. (2001). Range shifts and adaptive responses to quaternary climate change. Science 292, 673–679. doi: 10.1126/science.292.5517.673 11326089

[B15] Doyle J. J. Doyle J. L. (1987). A rapid DNA isolation procedure from small quantities of fresh leaf tissues. Phytochemical Bulletin 19, 11–15.

[B16] Drummond A. J. Ho S. Y. W. Phillips M. J. Rambaut A. (2006). Relaxed phylogenetics and dating with confidence. PloS Biol 4, e88. doi: 10.1371/journal.pbio.0040088 16683862 PMC1395354

[B17] Drummond A. J. Rambaut A. (2007). BEAST: Bayesian evolutionary analysis by sampling trees. BMC Evolutionary Biol 7, 214. doi: 10.1186/1471-2148-7-214 PMC2247476 17996036

[B18] Eckert C. G. Samis K. E. Lougheed S. C. (2008). Genetic variation across species’ geographical ranges: the central-marginal hypothesis and beyond. Mol. Ecology 17, 1170–1188. doi: 10.1111/j.1365-294x.2007.03659.x 18302683

[B19] Edgar R. C. (2004). MUSCLE: Multiple sequence alignment with high accuracy and high throughput. Nucleic Acids Res 32, 1792–1797. doi: 10.1093/nar/gkh340 15034147 PMC390337

[B20] Ehrenreich I. M. Purugganan M. D. (2008). Sequence variation of MicroRNAs and their binding sites in Arabidopsis. Plant Physiol 146, 1974–1982. doi: 10.1104/pp.108.116582 18305205 PMC2287364

[B21] El-Kassaby Y. A. (1991). Genetic variation within and among conifer populations: review and evaluation of methods. In: Biochemical markers in the population genetics of forest trees. Fineschi S. Malvolti M. E. Cannata F. Hattemer H. H., eds. (The Hague: SPA Academic). pp. 61–76.

[B22] Excoffier L. Lischer H. L. E. (2010). Arlequin suite ver 3.5: a new series of programs to perform population genetics analyses under Linux and Windows. Mol. Ecol. Resources 10, 564–567. doi: 10.1111/j.1755-0998.2010.02847.x 21565059

[B23] Fowler N. L. Dunlap D. W. (1986). Grassland vegetation of the eastern Edwards Plateau. Am. Midland Naturalist 115, 146–155. doi: 10.2307/2425844

[B24] Gehlbach F. R. (1991). The east-west transition zone of terrestrial vertebrates in Central Texas-A biogeographical analysis. Texas J. Science 43, 415–427. doi: 10.2307/3545274

[B25] Godbout J. Jaramillo-Correa J. P. Beaulieu J. Bousquet J. (2005). A mitochondrial DNA minisatellite reveals the postglacial history of jack pine (Pinus banksiana), a broad-range North American conifer. Mol. Ecology 14, 3497–3512. doi: 10.1111/j.1365-294x.2005.02674.x 16156818

[B26] Griffin S. R. Barrett S. (2004). Post-glacial history of *Trillium grandiflorum* (Melianthiaceae) in eastern North America: Inferences from phylogeography. Am. J. Botany 91, 465–473. doi: 10.3732/ajb.91.3.465 21653402

[B27] Hamrick J. L. Godt M. J. W. Sherman-Broyles S. L. (1992). Factors influencing levels of genetic diversity in woody plant species. New Forests 6, 95–124. doi: 10.1007/bf00120641

[B28] Hebert. W. Paul D. (2011). Phylogeography and postglacial dispersal of lake trout (Salvelinus namaycush) in north america. Can. J. Fisheries Aquat. Sci 55, 1010–1024. doi: 10.1139/cjfas-55-4-1010

[B29] Hewitt G. M. (1996). Some genetic consequences of ice ages, and their role in divergence and speciation. Biol. J. Linn. Society 58 247–276. doi: 10.1111/j.1095-8312.1996.tb01434.x

[B30] Hewitt G. M. (1999). Post-glacial re-colonization of European biota. Biol. J. Linn. Society 68, 87–112. doi: 10.1111/j.1095-8312.1999.tb01160.x

[B31] Hewitt G. M. (2000). The genetic legacy of the Quarternary ice ages. Nature 405, 907–913. doi: 10.1038/35016000 10879524

[B32] Jaramillo-Correa J. P. Beaulieu J. Bousquet J. (2004). Variation in mitochondrial DNA reveals multiple distant glacial refugia in black spruce (Picea mariana), a transcontinental North American conifer. Mol. Ecology 13, 2735–2747. doi: 10.1111/j.1365-294x.2004.02258.x 15315685

[B33] Jaramillo-Correa J. P. Beaulieu J. Khasa D. P. (2009). Inferring the past from the present phylogeographic structure of North American forest trees: seeing the forest for the genes. Can. J. For. Res 39, 286–307. doi: 10.1139/x08-181

[B34] Johnson K. A. Hendrix S. D. (2010). Wind pollination in the vitaceae: A case study of parthenocissus quinquefolia. J. Pollination Ecology 3, 12–18. doi: 10.26786/1920-7603(2010)3

[B35] Kim S. H. Cho M. S. Li P. Kim S. C. (2018). Phylogeography and ecological niche modeling reveal reduced genetic diversity and colonization patterns of skunk cabbage (Symplocarpus foetidus; Araceae) from glacial refugia in eastern North America. Front. Plant science 9. doi: 10.3389/fpls.2018.00648 PMC5972301 29872442

[B36] Kumar S. Stecher G. Tamura K. (2016). MEGA7: molecular evolutionary genetics analysis version 7.0 for bigger datasets. Mol. Biol. evolution 33, 1870–1874. doi: 10.1093/molbev/msw054 PMC8210823 27004904

[B37] Leigh J. W. Bryant. D. (2015). PopART: full-feature software for haplotype network construction. Methods Ecol Evol. 6 (9), 1110-1116. doi: 10.1111/2041-210X.12410

[B38] Li C. L. (1998). Vitaceae, flora republicae popularis sinica Vol. 48 (Beijing, China: Science Press).

[B39] Li P. Li M. Shi Y. Zhao Y. Wan Y. Fu C.. (2013). Phylogeography of North American herbaceous Smilax (Smilacaceae): combined AFLP and cpDNA data support a northern refugium in the Driftless Area. Am. J. Botany 100, 801–814. doi: 10.3732/ajb.1200250 23538874

[B40] Librado P. Rozas J. (2009). DnaSP v5: a software for comprehensive analysis of DNA polymorphism data. Bioinformatics 25, 1451–1452. doi: 10.1093/bioinformatics/btp187 19346325

[B41] Lu L. M. Wen J. Chen Z. (2012). A combined morphological and molecular phylogenetic analysis of Parthenocissus (Vitaceae) and taxonomic implications. Botanical J. Linn. Society 168, 43–63. doi: 10.1111/j.1095-8339.2011.01186.x

[B42] Ma Z. Y. Nie Z. L. Ren C. Liu X. Q. Wen J. (2021). Phylogenomic relationships and character evolution of the grape family (Vitaceae). Mol. Phylogenet. Evolution 154, 106948. doi: 10.1016/j.ympev.2020.106948 32866616

[B43] McLachlan J. S. Clark J. S. Manos P. S. (2005). Molecular indicators of tree migration capacity under rapid climate change. Ecology 86, 2088–2098. doi: 10.1890/04-1036

[B44] Mohn R. A. Oleas N. H. Smith A. B. Swift J. F. Yatskievych G. A. Edwards C. E. (2021). The phylogeographic history of a range disjunction in eastern North America: the role of post-glacial expansion into newly suitable habitat. Am. J. Botany 108, 1042–1057. doi: 10.1002/ajb2.1686 34156704

[B45] Moran C. Catterall C. P. Kanowski J. (2009). Reduced dispersal of native plant species as a consequence of the reduced abundance of frugivore species in fragmented rainforest. Biol. Conserv 142, 541–552. doi: 10.1016/j.biocon.2008.11.006

[B46] Morris A. B. Ickert-bond S. M. Brunson D. B. Soltis D. E. Soltis P. S. (2008). Phylogeographical structure and temporal complexity in American sweetgum (Liquidambar styraciflua; Altingiaceae). Mol. Ecology 17, 3889–3900. doi: 10.1111/j.1365-294X.2008.03875.x 18662227

[B47] Nadeau S. Godbout J. Lamothe M. Gros-Louis M. C. Lsabel N. Ritland K. (2015). Contrasting patterns of genetic diversity across the ranges of Pinus monticola and P. strobus: A comparison between eastern and western North American postglacial colonization histories. Am. J. Botany 102, 1342–1355. doi: 10.3732/ajb.1500160 26290557

[B48] Nei M. (1972). Genetic distance between populations. Am. Naturalist 106, 283–292. doi: 10.1086/282771

[B49] Nie Z. L. Sun H. Chen Z. D. Meng Y. Manchester S. R. Wen J. (2010). Molecular p,hylogeny and biogeographic diversification of Parthenocissus (Vitaceae) disjunct between Asia and North America. Am. J. Botany 97, 1342–1353. doi: 10.3732/ajb.1000085 21616887

[B50] O’Connell L. M. Mosseler A. Rajora O. P. (2007). Extensive long-distance pollen dispersal in a fragmented landscape maintains genetic diversity in white spruce. J. Heredity 98, 640–645. doi: 10.1093/jhered/esm089 17981919

[B51] Peakall R. Smouse P. E. (2006). Genalex 6: genetic analysis in Excel. Population genetic software for teaching and research. Mol. Ecol. Notes 6, 288–295. doi: 10.1111/j.1471-8286.2005.01155.x PMC3463245 22820204

[B52] Péros J. P. Cousins P. Launay A. Cubry P. Doligez A. (2021). Genetic diversity and population structure in Vitis species illustrate phylogeographic patterns in eastern North America. Mol. Ecology 30 (10), 2333-2348. doi: 10.1111/mec.15881 33710711

[B53] Perrier X. Jacquemoud-Collet J. P. (2006). DARwin software. Available online at: http://darwin.cirad.fr/ (Accessed October 26, 2020).

[B54] Peterson B. J. Graves W. R. (2016). Chloroplast phylogeography of Dirca palustris L. indicates populations near the glacial boundary at the Last Glacial Maximum in eastern North America. J. Biogeography 43, 314–327. doi: 10.1111/jbi.12621

[B55] Petit R. J. Duminil J. Fineschi S. Hampe A. Salvini D. Vendramin G. G. (2005). Comparative organization of chloroplast, mitochondrial and nuclear diversity in plant populations. Mol. Ecology 14, 689–701. doi: 10.1111/j.1365-294X.2004.02410.x 15723661

[B56] Pollefeys P. Bousquet J. (2003). Molecular genetic diversity of the French-American grapevine hybrids cultivated in North America. Genome/National Res. Council Canada = Génome/Conseil Natl. recherches Canada 46, 1037. doi: 10.1139/g03-076 14663522

[B57] Posada D. Crandall K. A. (1998). Modeltest: testing the model of DNA substitution. Bioinformatics 14 (9), 817–818. doi: 10.1093/bioinformatics/14.9.817 9918953

[B58] Prentice C. Bartlein P. J. Webb T. (1991). Vegetation and climate change in eastern North America since the Last Glacial Maximum. Ecology 72, 2038–2056. doi: 10.2307/1940469

[B59] Pritchard J. K. Stephens M. Donnelly P. (2000). Inference of population structure using multilocus genotype data. Genetics 155, 945–959. doi: 10.1093/genetics/155.2.945 10835412 PMC1461096

[B60] Robertson J. L. (1984). Pollination biology of parthenocissus quinquefolia (Vitaceae) in eastern north america. Am. J. Botany 71, 678–685. doi: 10.2307/2443361

[B61] Rogers A. R. Harpending H. (1992). Population growth makes waves in the distribution of pairwise genetic differences. Mol. Biol. Evolution.9 3), 552–569. doi: 10.1093/oxfordjournals.molbev.a040727 1316531

[B62] Ruiz-Sanchez E. Ornelas J. F. (2014). Phylogeography of Liquidambar styraciflua (Altingiaceae) in Mesoamerica: survivors of a Neogene widespread temperate forest (or cloud forest) in North America? Ecol. Evolution 4, 311–328. doi: 10.1002/ece3.938 PMC3936380 24634718

[B63] Schaal B. A. Hayworth D. A. Olsen K. M. Rauscher J. T. Smith W. A. (1998). Phylogeographic studies in plants: problems and prospects. Mol. Ecology 7, 465–474. doi: 10.1046/j.1365-294x.1998.0

[B64] Schaefer H. Heibl C. Renner S. S. (2009). Gourds afloat: a dated phylogeny reveals an Asian origin of the gourd family (Cucurbitaceae) and numerous oversea dispersal events. Proc. R. Soc. B: Biol. Sci 276, 843–851. doi: 10.1098/rspb.2008.1447 PMC2664369 19033142

[B65] Sewell M. M. Parks C. R. Chase M. W. (1996). Intraspecific chloroplast DNA variation and biogeography of North American Liriodendron L. (Magnoliaceae). Evolution 50, 1147–1154. doi: 10.1111/j.1558-5646.1996.tb02355.x 28565267

[B66] Slatkin M. Hudson R. R. (1991). Pairwise comparisons of mitochondrial DNA sequences in stable and exponentially growing populations. Genetics 129, 555–562. doi: 10.0000/PMID1743491 1743491 PMC1204643

[B67] Soejima A. Wen J. (2006). Phylogenetic analysis of the grape family (Vitaceae) based on three chloroplast markers. Am. J. Botany 93, 278–287. doi: 10.3732/ajb.93.2.278 21646189

[B68] Soltis D. E. Gitzendanner M. Strenge D. Soltis P. (1997). Chloroplast DNA intraspecific phylogeography of plants from the Pacific Northwest of North America. Plant Systematics Evolution 206, 353–373. doi: 10.1007/BF00987957

[B69] Soltis D. E. Kuzoff R. K. (1995). Discordance between nuclear and chloroplast phylogenies in the Heuchera group (Saxifragaceae). Evolution 49, 727–742. doi: 10.2307/2410326 28565145

[B70] Soltis D. E. Morris A. B. McLachan J. S. Manos P. S. Soltis P. S. (2006). Comparative phylogeography of unglaciated eastern North America. Mol. Ecology 15, 4261–4293. doi: 10.1111/j.1365-294X.2006.03061.x 17107465

[B71] Swenson N. G. Howard D. J. (2005). Clustering of contact zones, hybrid zones, and phylogeographic breaks in North America. Am. Naturalist 166, 581–591. doi: 10.2307/3491217 16224723

[B72] Swofford D. L. (2002). PAUP*. Phylogenetic analysis using parsimony (*and other methods). Version 4.0b10.mac version (Sunderland, Massachusetts, Sinauer Associates). doi: 10.1111/j.0014-3820.2002.tb00191.x

[B73] Taberlet P. Fumagalli L. Wust-Saucy A. G. Cosson J. F. (1998). Comparative phylogeography and postglacial colonization routes in Europe. Mol. ecology 7, 453–464. doi: 10.1046/j.1365-294x.1998.00289.x 9628000

[B74] Thompson L. M. Kevan P. G. (2012). Bird pollination in north american vines: A review with emphasis on parthenocissus quinquefolia. Ecol. Entomology 37, 321–330. doi: 10.1111/j.1365-2311.2012.01365

[B75] Tiffney B. H. Barghoorn E. S. (1976). Fruits and seeds of the brandon lignite. I. Vitaceae. Rev. Palaeobotany Palynology 22, 169–191. doi: 10.1016/0034-6667(76)90001-4

[B76] Tzedakis P. C. Frogley M. R. Heaton T. H. E. (2002). Duration of last interglacial conditions in northwestern Greece. Quaternary Res 58, 53–55. doi: 10.1006/qres.2002.2328

[B77] Wang J. Gao P. Kang M. Lowe A. J. Huang H. (2009). Refugia within refugia: the case study of a canopy tree (Eurycorymbus cavaleriei) in subtropical China. J. Biogeography 36, 2156–2164. doi: 10.1111/j.1365-2699.2009.02165.x

[B78] Wang X. R. Szmidt A. E. (2001). Molecular markers in population genetics of forest trees. Scandinavian J. For. Res 16, 199–220. doi: 10.1080/02827580118146

[B79] Wen J. (2007). “Vitaceae,” in The families and genera of vascular plants, vol. 9 . Ed. Kubitzki K. (Springer-Verlag, Berlin), 466–478.

[B80] Williams J. W. Shuman B. N. Webb T. Bartlein P. J. Leduc P. L. (2004). Late-quaternary vegetation dynamics in north america: scaling from taxa to biomes. Ecol. Monographs 74 (2), 309–334. doi: 10.1890/02-4045

[B81] Worth J. R. P. Jordan G. J. Marthick J. R. McKinnon G. E. Vaillancourt R. E. (2010). Chloroplast evidence for geographic stasis of the Australian bird-dispersed shrub Tasmannia lanceolata (Winteraceae). Mol. Ecology 19, 2949–2963. doi: 10.1111/j.1365-294X.2010.04725.x 20609080

[B82] Yu J. R. Niu Y. T. You Y. C. Cox C. J. Barrett R. L. Trias-Blasi A.. (2023). Integrated phylogenomic analyses unveil reticulate evolution in *Parthenocissus* (Vitaceae), highlighting speciation dynamics in the Himalayan-Hengduan Mountains. New Phytologist 238, 888–903. doi: 10.1111/nph.18580 36305244

[B83] Zachos J. Pagani M. Sloan L. Thomas E. Billups K. (2001). Trends, rhythms, and aberrations in global climate 65 Ma to present. Science 292, 686–693. doi: 10.1126/science.1059412 11326091

[B84] Zhao Y. P. Qi Z. C. Ma W. Dai Q. Li P. Cameron K. M.. (2013). Comparative phylogeography of the Smilax hispida group (Smilacaceae) in eastern Asia and North America-Implications for allopatric speciation, causes of diversity disparity, and origins of temperate elements in Mexico. Mol. Phylogenet. Evol 68, 300–311. doi: 10.1016/j.ympev.2013.03.025 23578597

[B85] Zinck J. W. R. Rajora O. P. (2016). Post-glacial phylogeography and evolution of a wide-ranging highly-exploited keystone forest tree, eastern white pine (Pinus strobus) in North America: single refugium, multiple routes. BMC Evolutionary Biol 16, 56–73. doi: 10.1186/s12862-016-0624-1 PMC4774161 26936598

