# Supplementary material for: Phylogeographic history of Parthenocissus (Vitaceae) in North America based on chloroplast and nuclear DNA sequences
Source: Front Plant Sci. 2025 Jun 25;16:1521784. doi: 10.3389/fpls.2025.1521784 (PMC12238048; doi:10.3389/fpls.2025.1521784)
Supplement: Supplementary file 1 [file DataSheet1.pdf]

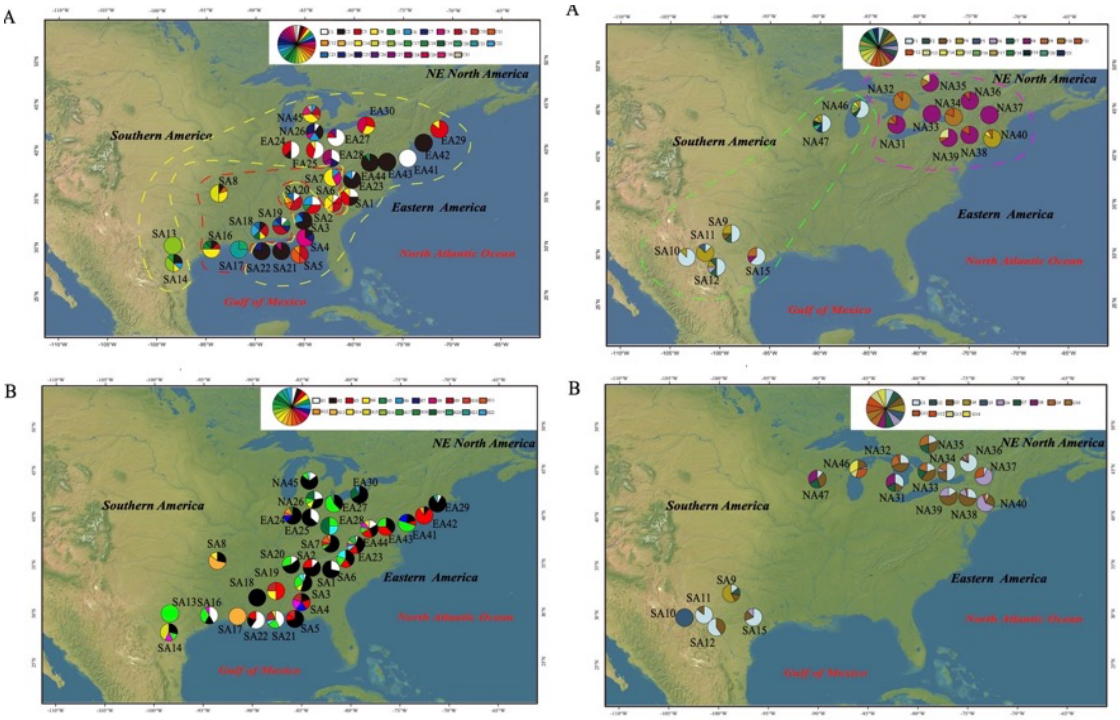

Fig. S1 Geographic distribution of cpDNA (A) and nrDNA (B) haplotypes detected in *P. quinquefolia* (left) and *P. vitacea* - *P. heptaphylla* (right) from North America. The dashed circles delimitate the two population groups detected by STRUCTURE analysis, comprising two large groups including southern group (light blue dashed line) and north group (orange dashed line).

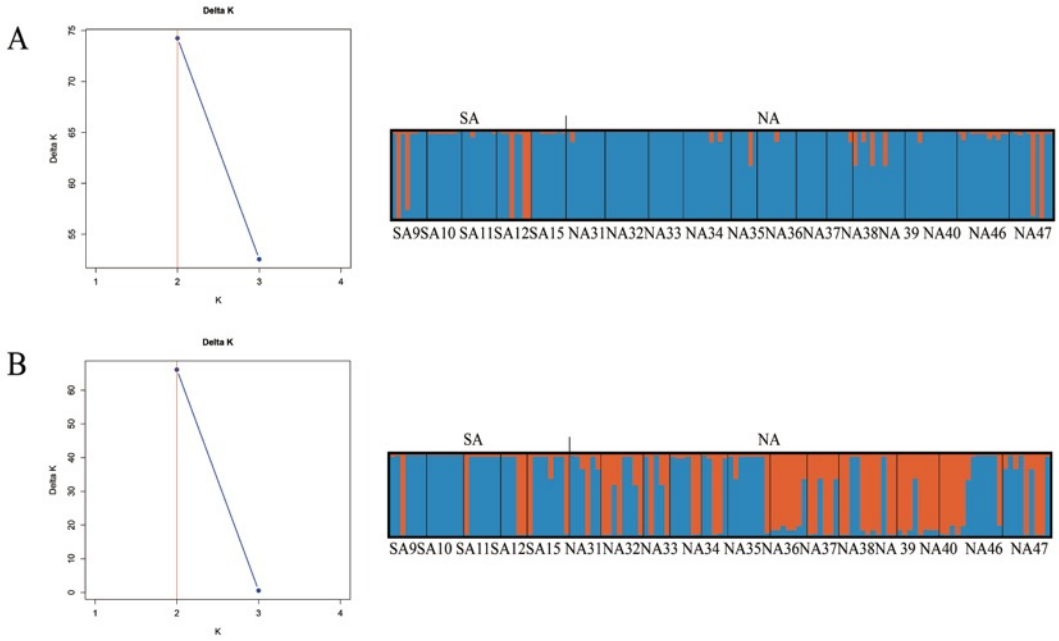

Fig. S2 STRUCTURE analysis for cpDNA and nrDNA data of 17 populations of *P. heptaphylla* and *P. vitacea* from North America. Number of clusters (K) was varied from two to 12 in 20 independent runs. (A) Histogram of the STRUCTURE analysis for the model with K=2 (showing the highest ΔK) in cpDNA data. (B) Histogram of the STRUCTURE analysis for the model with K=2 (showing the highest ΔK) in nrDNA.

Table S1 Geographic and haplotype characteristics of 17 *P. heptaphylla* and *P. vitacea* populations from North America surveyed for chloroplast (cp) DNA sequences and nuclear ribosome (nr) DNA variation

Pop	Locations	Latitude (°N)	Longitude (°E)	Chloroplast haplotype frequencies	Nuclear haplotype frequencies
SA9	Texas, Taylor	32.2370	99.8854	F1(4), F2(1), F3(1), F4(2)	G1(2), G2(2), G3(2), G4(8)
SA10	Texas, Jeff Davis	30.7052	104.2134	F1(7), F4(1)	G5(14)
SA11	Texas, Schleicher	30.9114	100.5846	F1(1), F4(6), F5(1)	G1(12), G3(2)
SA12	Texas, Kimble	30.2893	99.5244	F1(4), F2(1), F4(1), F6(1), F7(1)	G3(4), G1(6)
SA15	Texas, Blanco	30.3626	98.2776	F1(5), F8(1), F9(2)	G1(11), G3(3), G6(2)
NA31	Ontario, Grey	43.5387	80.2236	F8(7), F10(1), F11(1)	G1(4), G3(2), G7(2), G8(4)
NA32	Ontario, Grey	44.6140	80.7281	F10(9), F12(1)	G1(4), G3(7), G9(4), G10(1)
NA33	Ontario, Northumberland	44.3785	77.8674	F8(8)	G1(2), G3(4), G7(2), G9(2)
NA34	Ontario, Frontenac	44.7796	76.7225	F10(9), F11(2)	G1(6), G3(3), G6(1), G9(2)
NA35	Ontario, Glengarry	45.1749	74.8326	F8(4), F10(1), F13(1)	G1(2), G3(3)G7(2), G9(3)
NA36	Canada, Quebec	45.1741	73.1953	F8(8), F11(1)	G1(13), G3(1), G6(1), G9(1)
NA37	New Hampshire, Coos	44.6389	71.5421	F8(7)	G1(1), G6(9), G11(4)
NA38	Vermont, Bennington	42.8830	73.1544	F8(5), F11(1)	G1(2), G3(7), G6(3)
NA39	Massachusetts, Berkshire	42.3366	73.3324	F8(8), F11(1), F13(3)	G1(6), G3(12), G6(3), G11(1)
NA40	Massachusetts, Berkshire	42.2140	73.0979	F4(10), F11(1), F14(1)	G1(1), G3(4), G6(10), G12(1)
NA46	Michigan, Leelanau	44.2383	85.4007	F1(7), F4(1), F15(1), F16(1), F17(1), F18(1)	G3(5), G6(1), G11(7), G13(3), G14(8)
NA47	Wisconsin, West Salem	43.8969	91.0968	F1(5), F4(1), F17(1), F19(1), F20(1), F21(1)	G1(2), G3(6), G7(4), G8(6)
